# Functional Connectivity Abnormalities of Brain Regions with Structural Deficits in Young Adult Male Smokers

**DOI:** 10.3389/fnhum.2016.00494

**Published:** 2016-10-04

**Authors:** Limei Bu, Dahua Yu, Shaoping Su, Yao Ma, Karen M. von Deneen, Lin Luo, Jinquan Zhai, Bo Liu, Jiadong Cheng, Yanyan Guan, Yangding Li, Yanzhi Bi, Ting Xue, Xiaoqi Lu, Kai Yuan

**Affiliations:** ^1^Inner Mongolia Key Laboratory of Pattern Recognition and Intelligent Image Processing, School of Information Engineering, Inner Mongolia University of Science and TechnologyBaotou, People’s Republic of China; ^2^School of Life Science and Technology, Xidian UniversityXian, People’s Republic of China; ^3^Engineering Research Center of Molecular and NeuroImaging, Ministry of EducationPeople’s Republic of China; ^4^Department of Medical Imaging, The First Affiliated Hospital of Baotou Medical College, Inner Mongolia University of Science and TechnologyBaotou, People’s Republic of China

**Keywords:** smoking, voxel-based morphometry (VBM), resting state, functional connectivity, anterior cingulate cortex, putamen

## Abstract

Smoking is one of the most prevalent dependence disorders. Previous studies have detected structural and functional deficits in smokers. However, few studies focused on the changes of resting state functional connectivity (RSFC) of the brain regions with structural deficits in young adult smokers. Twenty-six young adult smokers and 26 well-matched healthy non-smokers participated in our study. Voxel-based morphometry (VBM) and RSFC were employed to investigate the structural and functional changes in young adult smokers. Compared with healthy non-smokers, young smokers showed increased gray matter (GM) volume in the left putamen and decreased GM volume in the left anterior cingulate cortex (ACC). Moreover, GM volume in the left ACC has a negative correlation trend with pack-years and GM volume in the left putamen was positively correlated with pack-years. The left ACC and putamen with abnormal volumes were chosen as the regions of interest (ROIs) for the RSFC analysis. We found that smokers showed increased RSFC between the left ACC and right amygdala and between the left putamen and right anterior insula. We revealed structural and functional deficits within the frontostriatal circuits in young smokers, which may shed new insights into the neural mechanisms of smoking.

## Introduction

Smoking related diseases cause more than 1 million yearly deaths in China (http://www.chinacdc.cn/). The latest national survey of smoking announced by the Chinese Center for Disease Control and Prevention in 2015, reported the smoking prevalence of people aged 15 years or older as 52.1% for males and 2.7% for females in China (http://www.chinacdc.cn/). Nowadays, China is the largest producer and consumer of tobacco in the world with 3.16 billion smokers including 14 million young smokers^1^ (Yu et al., 2011). The age period from late adolescence to adulthood is associated with the highest prevalence of cigarette smoking, which is also a time of continued brain development that may be affected by environmental perturbations such as nicotine exposure through cigarettes (Yu et al., 2016). Compared with older adult smokers, the frontal lobes of young adult smokers are undergoing a complex and prolonged developmental course from late adolescence through early adulthood which are involved in many higher-order cognitive functions (Taylor et al., 2014). Previous studies found that the white matter (WM) changes in smokers was nonlinear during adolescence to adulthood, which may suggest that developmental maturation of WM was stimulated by nicotine and the development of the frontal lobes was thus affected (Barnea-Goraly et al., 2005; Ashtari et al., 2007; Yu et al., 2016). Meanwhile, previous studies indicated that people who start smoking at an early age are more likely to become life-long smokers and are more susceptible to nicotine addiction than adults (Taioli and Wynder, 1991; O’Loughlin et al., 2003; White et al., 2009; Health and Services, 2012). Smoking during this special period may cause structural and functional changes in the brain and promote nicotine dependence for life (DeBry and Tiffany, 2008; Dwyer et al., 2009). Thus, it is extremely important to understand the neural mechanisms of smoking in young adult smokers.

Previous smoking studies revealed that the frontostriatal circuits played critical roles in reward and cognitive control pathways (Feil et al., 2010; Kober et al., 2010; Ma et al., 2010; Tomasi and Volkow, 2013; Motzkin et al., 2014; Jin et al., 2016; Yuan et al., 2016a, b). The structural and functional changes associated with the frontostriatal circuits in smokers have been investigated in previous studies (Bi et al., 2016; Feng et al., 2016; Li et al., 2016; Yuan et al., 2016a, b). Compared with healthy non-smokers, smokers showed decreased gray matter (GM) volumes in several brain regions, including the anterior cingulate cortex (ACC), prefrontal cortex (PFC), orbitofrontal cortex (OFC), cerebellum, thalamus and insula (Gallinat et al., 2006; Zhang et al., 2011; Kühn et al., 2012; Morales et al., 2012; Pan et al., 2013; Fritz et al., 2014) and increased GM volumes in the insula and putamen (Froeliger et al., 2010; Zhang et al., 2011; Franklin et al., 2014; Details in Supplementary Table S2). Moreover, resting state functional abnormalities in smokers were investigated at intra-regional and inter-regional levels. In more detail, regional changes in the ACC, insula, posterior cingulate cortex (PCC) and superior temporal gyrus (STG) were found by using the regional homogeneity (ReHo) analysis (Tang et al., 2012; Yu et al., 2013; Wu et al., 2015; Li et al., 2016). Increased fractional amplitude of low frequency fluctuation (fALFF) values in the caudate were revealed in our previous study (Feng et al., 2016). Decreased resting state functional connectivity (RSFC) between the ACC and insula as well as the bilateral caudate were reported in previous studies (Bi et al., 2016; Feng et al., 2016; Yuan et al., 2016b). Addicott et al. (2015) found that increased insula connectivity improved smoking abstinence (Details in Supplementary Table S1). However, few studies investigated the RSFC of brain regions with structural deficits in young smokers.

Therefore, we enrolled a relatively homogenous sample of participants in the current study to investigate structural changes between young smokers and healthy non-smokers. Then, the RSFC changes in the brain regions with structural deficits were analyzed. Given that previous smoking studies reported structural and functional changes in the frontostriatal circuits (Feng et al., 2016; Bi et al., 2016; Li et al., 2016; Yuan et al., 2016a, b), we hypothesized that structural and RSFC changes might be found within frontostriatal circuits and are correlated with smoking factors. We hope that our study may provide new insights into the neural mechanisms of smoking by combining structural and functional methods.

## Materials and Methods

All procedures were approved by the Medical Ethics Committee of the First Affiliated Hospital of Baotou Medical College, Inner Mongolia University of Science and Technology, and were conducted in accordance with the Declaration of Helsinki. All participants and their legal guardians in our study gave written informed consent after fully understanding the purposes of our study.

### Participants

Participants were recruited from local high schools and universities. The young smokers were screened according to the diagnostic criteria of nicotine dependence in the Diagnostic and Statistical Manual of Mental Disorders, Fifth Edition (DSM-V). Nicotine dependence levels were assessed with the Fagerström Test for Nicotine Dependence (FTND). All smokers used more than 10 Cigarettes Per Day (CPD) in the last 2 years and made no attempt to quit or undergo smoking abstinence longer than 3 months in the past year. Age-, education- and gender-matched healthy non-smokers were also enrolled. None of the healthy non-smokers had smoked more than five cigarettes in their lifetime. In order to avoid the effects of second hand smoke exposure, healthy non-smokers were recruited from non-smoking dormitories and neither of their parents smoked. Our analysis included several exclusion criteria, participants who: (1) have any physical illness assessed according to clinical evaluations and medical records including brain tumors, obstructive lung disease, hepatitis or epilepsy; (2) currently use any medications that may affect the normal activity of the brain; (3) drink alcohol or use drugs without restraint; (4) have a neurological disease; and (5) have claustrophobia. All of the participants were right-handed as measured by the Edinburgh Handedness Inventory.

Finally, we collected 26 young cigarette smokers and 26 age-, education- and gender-matched healthy non-smokers. The expiratory carbon monoxide (CO) levels of all participants were measured using the Smokerlyzer system (Bedfont Scientific Ltd., Rochester, UK). CO level in expired air was verified as ≥10 ppm in smokers and ≤3 ppm in healthy non-smokers. Detailed demographic characteristics are given in Table 1.

**Table 1:** **Demographic characteristics of young smokers and healthy non-smokers in the present study**.

	Smokers (*n* = 26)	Non-smokers (*n* = 26)
Male/female	26/0	26/0
Age (years)	21.42 ± 1.73	20.58 ± 1.47
Age range (years)	19–26	19–25
Levels of Education	13.92 ± 0.83	13.65 ± 0.68
Cigarettes Per Day (CPD)	15.04 ± 4.82	-
Age at Start of Smoking	14.96 ± 3.26	-
Years of Smoking	4.27 ± 2.44	-
Pack-Years	3.55 ± 2.97	-
FTND	4.42 ± 2.20	-

*Values are expressed as means ± standard deviations. FTND, Fagerström Test for Nicotine Dependence. Pack-years: smoking years × Daily consumption/20*.

### Data Acquisition

All image data were acquired on a 3T Philips scanner (Achieva; Philips Medical Systems, Best, Netherlands) at the First Affiliated Hospital of Baotou Medical College, Inner Mongolia University of Science and Technology, Baotou, China. The heads of the subjects were restrained with foam pads and positioned carefully with comfortable support. To reduce scanner noise, ear plugs were used during the scan. A high-resolution T1 structural image was acquired using a magnetization prepared rapid acquisition gradient echo (MPRAGE) pulse sequence with a voxel size of 1 mm^3^ (repetition time (TR) = 8.4 ms; echo time (TE) = 3.8 ms; data matrix = 240 × 240; slices = 176; field of view (FOV) = 240 mm^2^ × 240 mm^2^). Then, the resting state functional images were obtained with an echo-planar imaging (EPI) sequence (32 contiguous slices with slice thickness = 5 mm, TR = 2000 ms, TE = 30 ms, flip angle = 90°, FOV = 224 mm^2^ × 224 mm^2^, data matrix = 64 × 64 and total volumes = 185). Subjects were instructed to stay awake, keep their eyes closed and not think about anything during the whole scan. After the scan, all of the participants were asked whether they remained awake during the whole procedure. Two expert radiologists examined the images of all participants to exclude any clinically silent lesions.

### Data Analysis

T1 images were preprocessed by using Statistical Parametric Mapping 8 (SPM8; Wellcome Department of Cognitive Neurology) and VBM8 toolbox (University of Jena, Department of Psychiatry) implemented in Matlab 7.12.0. Firstly, the T1 images were segmented into three parts including GM, WM, and cerebrospinal fluid (CSF). Then, by using affine registration, the segmented images were transferred into the stereotactic Montreal Neurological Institute (MNI) space. Subsequently, by using high-dimensional DARTEL normalization and Jacobian determinants, the segmented and registered images were normalized and modulated. Finally, GM images were smoothed using an 8 mm full width at half maximum (FWHM) Gaussian kernel (Froeliger et al., 2010; Fritz et al., 2014). In order to examine the difference in GM volume between young smokers and healthy non-smokers, a two-sample *t*-test with age as a covariate was conducted in SPM (family-wise error (FWE) correction, *p* < 0.05).

To investigate the relationship between GM volume findings and smoking factors, the regions showing altered volumes in young smokers compared with healthy non-smokers were selected and defined as the regions of interest (ROIs). Then, the mean volumes of these ROIs were extracted by using the Resting State fMRI Data Analysis Toolkit (REST, V1.8^2^; Song et al., 2011). In older to eliminate the influence of age, the partial correlation analysis was analyzed by using the IBM SPSS statistics (version 20.0, SPSS Inc, Chicago, IL, USA).

The resting-state fMRI data were preprocessed by using Data Processing Assistant for Resting-State fMRI (DPARSF, V3.1^3^), which was based on SPM8 and REST V1.8 software (Song et al., 2011). Preprocessing included the following steps. First, remove the first 10 time points of the scanning sessions due to the scanner stability and participants’ adaptation to the scanning environment. Second, perform slice timing due to the acquisition delay between slices. Third, correct for head realignment by using a rigid body transformation. Fourth, transfer into stereotactic MNI space with a resampling voxel size of 3 mm × 3 mm × 3 mm. Finally, a 6 mm FWHM Gaussian kernel was used to smooth the images. Additionally, subjects with head motion exceeding 1 mm of movement or 1° rotation in any direction were excluded from the current study (Feng et al., 2016).

The regions showing altered volumes in smokers compared with healthy non-smokers were defined as ROIs. The averaged fMRI time series for total voxels of each ROI was considered as the reference time series. RSFC analysis for each ROI was conducted between the averaged fMRI time series and every other voxel in the whole-brain for each individual’s preprocessed data in a voxel-wise measure by the REST V1.8 software (Song et al., 2011). The resultant *r* value maps were transformed to approximate Gaussian distribution with a Fisher’s *z* transformation. Two-sample *t*-test was applied to compare the RSFC differences between groups in SPM8 (*p* < 0.05, FWE corrected).

## Results

### VBM Results

**Figure 1:**
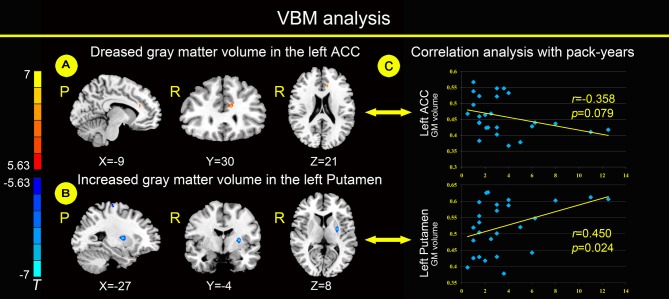
**Compared with healthy non-smokers (*P* < 0.05, Family-wise error (FWE) corrected), young smokers showed significantly decreased gray matter (GM) volume in the left anterior cingulate cortex (ACC; A) and increased GM volume in the left putamen (B)**. The GM volume of the left ACC has a negative correlation trend with pack-years, but not a significant correlation. The GM volume of the left putamen was positively correlated with pack-years **(C)**. Pack-years = smoking years × daily consumption/20.

Relative to healthy non-smokers, young smokers showed a significantly decreased GM volume in the left ACC and increased GM volume in the left putamen (Figures 1A,B). The GM volume in the left ACC had a negative correlation trend with pack-years (Figure 1C; *r* = −0.358, *p* = 0.079), but not a significant correlation. The GM volume in the left putamen was positively correlated with pack-years (Figure 1C; *r* = 0.450, *p* = 0.024).

### Resting-State Results

**Figure 2:**
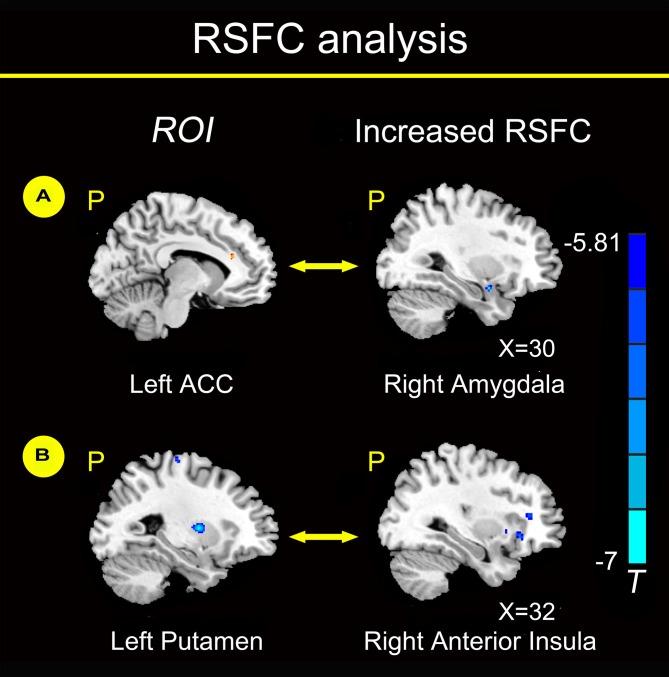
**Functional connectivity analysis (*P* < 0.05, FWE corrected)**. We chose the left ACC as the region of interest (ROI) and found increased functional connectivity between the left ACC and right amygdala in young smokers compared to healthy non-smokers **(A)**. We chose the left putamen as the ROI and found increased functional connectivity between the left putamen and right anterior insula in young smokers compared to healthy non-smokers **(B)**.

Compared with healthy non-smokers, young smokers showed increased functional connectivity between the left ACC and right amygdala and between the left putamen and right anterior insula (Figure 2). No regions showed significant decreased functional connectivity with the left ACC and left putamen in young smokers.

## Discussion

People who start smoking early are more likely to become life-long smokers. The study of smokers during adolescence and early adulthood may improve our understanding of the neural mechanisms of smoking. In the present study, we investigated structural and functional changes in young smokers compared with healthy non-smokers by combining Voxel-based morphometry (VBM) and RSFC methods. VBM provides local structural changes of GM volume between young adult smokers and nonsmokers (Ashburner and Friston, 2000). Moreover, RSFC provides inter-regional functional changes between brain regions during resting state (Bi et al., 2016). By combining VBM and RSFC methods, functional connectivity changes in brain regions with structural deficits in young adult male smokers were investigated, which may provide more evidence about the neural mechanisms of smoking. Compared with healthy non-smokers, young smokers showed decreased GM volumes in the left ACC and increased GM volumes in the left putamen. The GM volume in the left ACC had a negative correlation trend with pack-years, but not a significant correlation. The GM volume in the left putamen was positively correlated with pack-years. Moreover, smokers showed increased RSFC between the left ACC and right amygdala and between the left putamen and right anterior insula.

Structural differences within frontostriatal circuits between young smokers and healthy non-smokers were reported in previous studies (Froeliger et al., 2010; Yu et al., 2011; Pan et al., 2013; Fritz et al., 2014; Li et al., 2015). Consistent with previous findings in smoking (Froeliger et al., 2010; Pan et al., 2013; Franklin et al., 2014; Fritz et al., 2014), young smokers showed a decreased GM volume in the left ACC and increased GM volume in the left putamen (Figures 1A,B). As a key part of frontostriatal circuits, the ACC was related to cognitive and emotional processing based on evidence from electrical stimulation, electroencephalography, positron emission tomography (PET), functional MRI (fMRI) and lesion studies (Bush et al., 2000; Allman et al., 2001). The activation of the ACC was correlated with craving for smoking, which may be related to the urge of smoking (Kober et al., 2010; Li et al., 2013). Decreased activation of the ACC was associated with resisting cravings in response to cigarette cues and was involved in emotional regulation dysfunction in smokers (Brody et al., 2007; Fritz et al., 2014).

The putamen is a critical part of the reward pathway (Das et al., 2012). Hyperactivity of the putamen in smokers was reported when exposed to environmental cues triggering craving (Brody et al., 2002; McClernon et al., 2005; Yuan et al., 2016b). Particularly, the putamen is critical for the development of nicotine addiction, and a high concentration of nicotinic acetylcholine receptors (nAChR) in the putamen makes it a potential target for nicotine (Das et al., 2012). Moreover, the GM volume in the left ACC has a negative correlation trend with pack-years, but not a significant correlation. The GM volume in the left putamen was positively correlated with pack-years (Figure 1C), which may be a biomarker of the cumulative effect of smoking.

Furthermore, RSFC changes were investigated in brain regions with structural deficits in our study. Compared with healthy non-smokers, young smokers showed increased functional connectivity between the left ACC and right amygdala and between the left putamen and right anterior insula (Figure 2). RSFC may provide inter-regional functional changes by measuring the temporal correlation strength between the intrinsic fluctuations observed in spatially distinct brain regions during resting state (Biswal et al., 1995; Fox and Raichle, 2007). Previous studies reported changes in RSFC between the ACC-striatum and ACC-insula, which may serve as a circuit-level biomarker for smoking (Hong et al., 2009; Bi et al., 2016; Feng et al., 2016; Yuan et al., 2016b). The amygdala is a key node of the dopamine circuits in addiction (Goldstein and Volkow, 2002). The amygdala is involved in the association of discrete stimuli that respond to emotionally evocative stimuli, which is a particularly important topic in smoking (Everitt and Robbins, 2005). Chase et al. (2011) found that the involvement of the amygdala was associated with the development of nicotine dependence. Compared to neutral cues, amygdala reactivity was higher to smoking-related cues, and the absolute regional cerebral blood flow (rCBF) changes in the bilateral amygdala were positively correlated with abstinence-induced craving (Toselli et al., 2015). Young smokers showed significantly decreased GM volume in the left ACC, and the RSFC between the left ACC and right amygdala was increased. A possible explanation might be associated with compensation mechanism of neurons, which might maintain a relatively normal cognitive function by increasing the RSFC of the ACC-amygdala circuit. Previous studies confirmed deficits in cognitive processing supported by the PFC, which may be compensated by increased activity in that particular compensatory region (Voytek et al., 2010; Hou et al., 2016; Sitek et al., 2016). We hypothesize that this compensatory mechanism might explain the brain activity which forces the amygdala into overdrive and therefore increases craving.

Additionally, we found increased functional connectivity between the left putamen and right anterior insula in young adult smokers. Naqvi et al. (2007) reported that damage to the insula disrupted smoking behavior in stroke patients, which supports a critical role for the insula in the maintenance of smoking addiction. Previous studies found that increased insula connectivity might be associated with improved smoking abstinence (Janes et al., 2010; Addicott et al., 2015). The anterior insula is a key component of the salience network (SN) and maintains a variety of foundational capacities fundamental to cognitive function (Dosenbach et al., 2007; Larson-Prior et al., 2009). Meanwhile, the anterior insula is structurally and functionally connected to the ACC, dorsal lateral PFC (DLPFC), ventromedial PFC (VMPFC), amygdala and striatum, because these regions are commonly implicated in craving and in the ability to control the urge to smoke (Mesulam and Mufson, 1982; Kober et al., 2010; Deen et al., 2011; Li et al., 2013). For instance, young smokers showed decreased RSFC between the anterior insula and ACC, VMPFC, amygdala, DLPFC and dorsal striatum (Bi et al., 2016). Particularly, the insula has been proven to have a high density of nAChRs within the human cerebral cortex (Picard et al., 2013). Therefore, the insula may be more vulnerable to the addictive effects of nicotine, which are related to aberrant processing of cognitive control and craving in smokers. The inter-regional changes in the frontostriatal circuits affected the response to the addictive substance, which may maintain the compulsiveness to nicotine and form a vicious cycle of nicotine addiction (Goldstein and Volkow, 2002). The changes in the RSFC between the ACC-amygdala and putamen-insula may be modified by smoking and serve as a circuit-level biomarker with structural deficits.

### Limitations

The Chinese Center for Disease Control and Prevention found that men have about 20 times the incidence of smoking compared to women^4^. Taking into account the social acceptance of female smokers, they were reluctant to provide relevant information and participate in research. Considering the clear gender effect of smokers’ GM changes (Kühn et al., 2012; Franklin et al., 2014), an important limitation of the present study is only including young male subjects. It is unclear whether our findings generalize to young female subjects. Thus, we need further studies to investigate the gender effect in smokers.

## Conclusion

In the present study, we found structural changes in the ACC and putamen accompanied by RSFC changes. The findings may provide new insights into the structural and functional changes within the frontostriatal circuits of young smokers. We hope the findings may improve the understanding of neural mechanisms in smoking.

## Author Contributions

LB, DY, KY and XL conceived and designed the experiments; KY, SS, YM, LL, JZ and BL performed the experiments; DY, KY, LB, YG, YL and YB analyzed the data; LB, DY and KY wrote the article; KMD and TX provided critical revision of the manuscript for important intellectual content. All authors critically reviewed the content and approved the final version for publication.

## Funding

This article is supported by the Project for the National Key Basic Research and Development Program (973) under Grant Nos. 2014CB543203, 2011CB707700, 2012CB518501, the National Natural Science Foundation of China under Grant Nos. 81301281, 81571753, 81571751, 81401488, 61179019, 81401478, 81271644, 81271546, 81271549, 81470816, 81471737, the Natural Science Basic Research Plan in Shaanxi Province of China under Grant No. 2014JQ4118, the Fundamental Research Funds for the Central Universities under the Grant Nos. JB151204, JB121405, the Natural Science Foundation of Inner Mongolia under Grant No. 2014BS0610, 2015MS0604, the Innovation Fund Project of Inner Mongolia University of Science and Technology Nos. 2015QNGG03, 2014QDL002, and the General Financial Grant from the China Post-doctoral Science Foundation under Grant No. 2014M552416.

## Supplementary Material

The Supplementary Material for this article can be found online at: http://journal.frontiersin.org/article/10.3389/fnhum.2016.00494

## Conflict of Interest Statement

The authors declare that the research was conducted in the absence of any commercial or financial relationships that could be construed as a potential conflict of interest.

## Footnotes

^1^http://www.chinacdc.cn/

^2^http://restfmri.net/forum/REST_V1.8

^3^http://www.restfmri.net

^4^http://www.chinacdc.cn/

## References

[B1] Addicott M. A. Sweitzer M. M. Froeliger B. Rose J. E. McClernon F. J. (2015). Increased functional connectivity in an insula-based network is associated with improved smoking cessation outcomes. Neuropsychopharmacology 40, 2648–2656. 10.1038/npp.2015.114 25895453 PMC4569957

[B2] Allman J. M. Hakeem A. Erwin J. M. Nimchinsky E. Hof P. (2001). The anterior cingulate cortex. The evolution of an interface between emotion and cognition. Ann. N Y Acad. Sci. 935, 107–117. 10.1111/j.1749-6632.2001.tb03476.x 11411161

[B3] Ashburner J. Friston K. J. (2000). Voxel-based morphometry–the methods. Neuroimage 11, 805–821. 10.1006/nimg.2000.0582 10860804

[B4] Ashtari M. Cervellione K. L. Hasan K. M. Wu J. McIlree C. Kester H.. (2007). White matter development during late adolescence in healthy males: a cross-sectional diffusion tensor imaging study. Neuroimage 35, 501–510. 10.1016/j.neuroimage.2006.10.047 17258911

[B5] Barnea-Goraly N. Menon V. Eckert M. Tamm L. Bammer R. Karchemskiy A.. (2005). White matter development during childhood and adolescence: a cross-sectional diffusion tensor imaging study. Cereb. Cortex 15, 1848–1854. 10.1093/cercor/bhi062 15758200

[B6] Bi Y. Yuan K. Guan Y. Cheng J. Zhang Y. Li Y.. (2016). Altered resting state functional connectivity of anterior insula in young smokers. Brain Imaging Behav. [Epub ahead of print]. 10.1007/s11682-016-9511-z 26843002

[B7] Biswal B. Yetkin F. Z. Haughton V. M. Hyde J. S. (1995). Functional connectivity in the motor cortex of resting human brain using echo–planar MRI. Magn. Reson. Med. 34, 537–541. 10.1002/mrm.1910340409 8524021

[B8] Brody A. L. Mandelkern M. A. London E. D. Childress A. R. Lee G. S. Bota R. G.. (2002). Brain metabolic changes during cigarette craving. Arch. Gen. Psychiatry 59, 1162–1172. 10.1001/archpsyc.59.12.1162 12470133

[B9] Brody A. L. Mandelkern M. A. Olmstead R. E. Jou J. Tiongson E. Allen V.. (2007). Neural substrates of resisting craving during cigarette cue exposure. Biol. Psychiatry 62, 642–651. 10.1016/j.biopsych.2006.10.026 17217932 PMC1992815

[B10] Bush G. Luu P. Posner M. I. (2000). Cognitive and emotional influences in anterior cingulate cortex. Trends Cogn. Sci. 4, 215–222. 10.1016/s1364-6613(00)01483-2 10827444

[B11] Chase H. W. Eickhoff S. B. Laird A. R. Hogarth L. (2011). The neural basis of drug stimulus processing and craving: an activation likelihood estimation meta-analysis. Biol. Psychiatry 70, 785–793. 10.1016/j.biopsych.2011.05.025 21757184 PMC4827617

[B12] Das D. Cherbuin N. Anstey K. J. Sachdev P. S. Easteal S. (2012). Lifetime cigarette smoking is associated with striatal volume measures. Addict. Biol. 17, 817–825. 10.1111/j.1369-1600.2010.00301.x 21392170

[B50] DeBry S. C. Tiffany S. T. (2008). Tobacco-induced neurotoxicity of adolescent cognitive development (TINACD): a proposed model for the development of impulsivity in nicotine dependence. Nicotine Tob. Res. 10, 11–25. 10.1080/14622200701767811 18188741

[B13] Deen B. Pitskel N. B. Pelphrey K. A. (2011). Three systems of insular functional connectivity identified with cluster analysis. Cereb. Cortex 21, 1498–1506. 10.1093/cercor/bhq186 21097516 PMC3116731

[B14] Dosenbach N. U. Fair D. A. Miezin F. M. Cohen A. L. Wenger K. K. Dosenbach R. A.. (2007). Distinct brain networks for adaptive and stable task control in humans. Proc. Natl. Acad. Sci. U S A 104, 11073–11078. 10.1073/pnas.0704320104 17576922 PMC1904171

[B15] Dwyer J. B. McQuown S. C. Leslie F. M. (2009). The dynamic effects of nicotine on the developing brain. Pharmacol. Ther. 122, 125–139. 10.1016/j.pharmthera.2009.02.003 19268688 PMC2746456

[B16] Everitt B. J. Robbins T. W. (2005). Neural systems of reinforcement for drug addiction: from actions to habits to compulsion. Nat. Neurosci. 8, 1481–1489. 10.1038/nn1579 16251991

[B17] Feil J. Sheppard D. Fitzgerald P. B. Yücel M. Lubman D. I. Bradshaw J. L. (2010). Addiction, compulsive drug seeking and the role of frontostriatal mechanisms in regulating inhibitory control. Neurosci. Biobehav. Rev. 35, 248–275. 10.1016/j.neubiorev.2010.03.001 20223263

[B18] Feng D. Yuan K. Li Y. Cai C. Yin J. Bi Y.. (2016). Intra-regional and inter-regional abnormalities and cognitive control deficits in young adult smokers. Brain Imaging Behav. 10, 506–516. 10.1007/s11682-015-9427-z 26164168

[B19] Fox M. D. Raichle M. E. (2007). Spontaneous fluctuations in brain activity observed with functional magnetic resonance imaging. Nat. Rev. Neurosci. 8, 700–711. 10.1038/nrn2201 17704812

[B20] Franklin T. R. Wetherill R. R. Jagannathan K. Johnson B. Mumma J. Hager N.. (2014). The effects of chronic cigarette smoking on gray matter volume: influence of sex. PLoS One 9:e104102. 10.1371/journal.pone.0104102 25090480 PMC4121321

[B21] Fritz H.-C. Wittfeld K. Schmidt C. O. Domin M. Grabe H. J. Hegenscheid K.. (2014). Current smoking and reduced gray matter volume—a voxel-based morphometry study. Neuropsychopharmacology 39, 2594–2600. 10.1038/npp.2014.112 24832823 PMC4207339

[B22] Froeliger B. Kozink R. V. Rose J. E. Behm F. M. Salley A. N. McClernon F. J. (2010). Hippocampal and striatal gray matter volume are associated with a smoking cessation treatment outcome: results of an exploratory voxel-based morphometric analysis. Psychopharmacology (Berl) 210, 577–583. 10.1007/s00213-010-1862-3 20424827

[B23] Gallinat J. Meisenzahl E. Jacobsen L. K. Kalus P. Bierbrauer J. Kienast T.. (2006). Smoking and structural brain deficits: a volumetric MR investigation. Eur. J. Neurosci. 24, 1744–1750. 10.1111/j.1460-9568.2006.05050.x 17004938

[B24] Goldstein R. Z. Volkow N. D. (2002). Drug addiction and its underlying neurobiological basis: neuroimaging evidence for the involvement of the frontal cortex. Am. J. Psychiatry 159, 1642–1652. 10.1176/appi.ajp.159.10.1642 12359667 PMC1201373

[B25] Health U. D. O. Services H. (2012). Preventing Tobacco Use Among Youth and Young Adults: A Report of the Surgeon General (Vol. 3). Atlanta, GA: US Department of Health and Human Services, Centers for Disease Control and Prevention, National Center for Chronic Disease Prevention and Health Promotion, Office on Smoking and Health.

[B26] Hong L. E. Gu H. Yang Y. Ross T. J. Salmeron B. J. Buchholz B.. (2009). Association of nicotine addiction and nicotine’s actions with separate cingulate cortex functional circuits. Arch. Gen. Psychiatry 66, 431–441. 10.1001/archgenpsychiatry.2009.2 19349313 PMC2774753

[B27] Hou Y. Yang J. Luo C. Ou R. Song W. Liu W.. (2016). Patterns of striatal functional connectivity differ in early and late onset Parkinson’s disease. J. Neurol. [Epub ahead of print]. 10.1007/s00415-016-8211-3 27394147

[B28] Janes A. C. Pizzagalli D. A. Richardt S. deB Frederick B. Chuzi S. Pachas G.. (2010). Brain reactivity to smoking cues prior to smoking cessation predicts ability to maintain tobacco abstinence. Biol. Psychiatry 67, 722–729. 10.1016/j.biopsych.2009.12.034 20172508 PMC2954596

[B29] Jin C. Zhang T. Cai C. Bi Y. Li Y. Yu D.. (2016). Abnormal prefrontal cortex resting state functional connectivity and severity of internet gaming disorder. Brain Imaging Behav. 10, 719–729. 10.1007/s11682-015-9439-8 26311395

[B30] Kober H. Mende-Siedlecki P. Kross E. F. Weber J. Mischel W. Hart C. L.. (2010). Prefrontal-striatal pathway underlies cognitive regulation of craving. Proc. Natl. Acad. Sci. U S A 107, 14811–14816. 10.1073/pnas.1007779107 20679212 PMC2930456

[B31] Kühn S. Romanowski A. Schilling C. Mobascher A. Warbrick T. Winterer G.. (2012). Brain grey matter deficits in smokers: focus on the cerebellum. Brain Struct. Funct. 217, 517–522. 10.1007/s00429-011-0346-5 21909705

[B32] Larson-Prior L. J. Zempel J. M. Nolan T. S. Prior F. W. Snyder A. Z. Raichle M. E. (2009). Cortical network functional connectivity in the descent to sleep. Proc. Natl. Acad. Sci. U S A 106, 4489–4494. 10.1073/pnas.0900924106 19255447 PMC2657465

[B33] Li X. Hartwell K. J. Borckardt J. Prisciandaro J. J. Saladin M. E. Morgan P. S.. (2013). Volitional reduction of anterior cingulate cortex activity produces decreased cue craving in smoking cessation: a preliminary real–time fMRI study. Addict. Biol. 18, 739–748. 10.1111/j.1369-1600.2012.00449.x 22458676 PMC3389595

[B34] Li Y. Yuan K. Bi Y. Guan Y. Cheng J. Zhang Y.. (2016). Neural correlates of 12-h abstinence-induced craving in young adult smokers: a resting-state study. Brain Imaging Behav. [Epub ahead of print]. 10.1007/s11682-016-9544-3 26995747

[B35] Li Y. Yuan K. Cai C. Feng D. Yin J. Bi Y.. (2015). Reduced frontal cortical thickness and increased caudate volume within fronto-striatal circuits in young adult smokers. Drug Alcohol Depend. 151, 211–219. 10.1016/j.drugalcdep.2015.03.023 25865908

[B36] Ma N. Liu Y. Li N. Wang C.-X. Zhang H. Jiang X.-F.. (2010). Addiction related alteration in resting-state brain connectivity. Neuroimage 49, 738–744. 10.1016/j.neuroimage.2009.08.037 19703568 PMC2764798

[B37] McClernon F. J. Hiott F. B. Huettel S. A. Rose J. E. (2005). Abstinence-induced changes in self-report craving correlate with event-related FMRI responses to smoking cues. Neuropsychopharmacology 30, 1940–1947. 10.1038/sj.npp.1300780 15920499 PMC1571136

[B38] Mesulam M. Mufson E. J. (1982). Insula of the old world monkey. III: efferent cortical output and comments on function. J. Comp. Neurol. 212, 38–52. 10.1002/cne.902120104 7174907

[B39] Morales A. M. Lee B. Hellemann G. O’Neill J. London E. D. (2012). Gray-matter volume in methamphetamine dependence: cigarette smoking and changes with abstinence from methamphetamine. Drug Alcohol Depend. 125, 230–238. 10.1016/j.drugalcdep.2012.02.017 22445480 PMC3427723

[B40] Motzkin J. C. Baskin–Sommers A. Newman J. P. Kiehl K. A. Koenigs M. (2014). Neural correlates of substance abuse: reduced functional connectivity between areas underlying reward and cognitive control. Hum. Brain Mapp. 35, 4282–4292. 10.1002/hbm.22474 24510765 PMC4107096

[B41] Naqvi N. H. Rudrauf D. Damasio H. Bechara A. (2007). Damage to the insula disrupts addiction to cigarette smoking. Science 315, 531–534. 10.1126/science.1135926 17255515 PMC3698854

[B42] O’Loughlin J. DiFranza J. Tyndale R. F. Meshefedjian G. McMillan-Davey E. Clarke P. B.. (2003). Nicotine-dependence symptoms are associated with smoking frequency in adolescents. Am. J. Prev. Med. 25, 219–225. 10.1016/s0749-3797(03)00198-3 14507528

[B43] Pan P. Shi H. Zhong J. Xiao P. Shen Y. Wu L.. (2013). Chronic smoking and brain gray matter changes: evidence from meta-analysis of voxel-based morphometry studies. Neurol. Sci. 34, 813–817. 10.1007/s10072-012-1256-x 23207549

[B44] Picard F. Sadaghiani S. Leroy C. Courvoisier D. S. Maroy R. Bottlaender M. (2013). High density of nicotinic receptors in the cingulo-insular network. Neuroimage 79, 42–51. 10.1016/j.neuroimage.2013.04.074 23631995

[B45] Sitek K. R. Cai S. Beal D. S. Perkell J. S. Guenther F. H. Ghosh S. S. (2016). Decreased cerebellar-orbitofrontal connectivity correlates with stuttering severity: whole-brain functional and structural connectivity associations with persistent developmental stuttering. Front. Hum. Neurosci. 10:190. 10.3389/fnhum.2016.00190 27199712 PMC4855981

[B46] Song X.-W. Dong Z.-Y. Long X.-Y. Li S.-F. Zuo X.-N. Zhu C.-Z.. (2011). REST: a toolkit for resting-state functional magnetic resonance imaging data processing. PLoS One 6:e25031. 10.1371/journal.pone.0025031 21949842 PMC3176805

[B47] Taioli E. Wynder E. L. (1991). Effect of the age at which smoking begins on frequency of smoking in adulthood. N. Engl. J. Med. 325, 968–969. 10.1056/nejm199109263251318 1881424

[B48] Tang J. Liao Y. Deng Q. Liu T. Chen X. Wang X.. (2012). Altered spontaneous activity in young chronic cigarette smokers revealed by regional homogeneity. Behav. Brain Funct. 8:44. 10.1186/1744-9081-8-44 22913365 PMC3511796

[B49] Taylor M. J. Doesburg S. M. Pang E. W. (2014). Neuromagnetic vistas into typical and atypical development of frontal lobe functions. Front. Hum. Neurosci. 8:453. 10.3389/fnhum.2014.00453 24994980 PMC4061489

[B51] Tomasi D. Volkow N. D. (2013). Striatocortical pathway dysfunction in addiction and obesity: differences and similarities. Crit. Rev. Biochem. Mol. Biol. 48, 1–19. 10.3109/10409238.2012.735642 23173916 PMC3557663

[B52] Toselli F. Booth Depaz I. M. Worrall S. Etheridge N. Dodd P. R. Wilce P. A.. (2015). Expression of CYP2E1 and CYP2U1 proteins in amygdala and prefrontal cortex: influence of alcoholism and smoking. Alcohol. Clin. Exp. Res. 39, 790–797. 10.1111/acer.12697 25872594

[B53] Voytek B. Davis M. Yago E. Barceló F. Vogel E. K. Knight R. T. (2010). Dynamic neuroplasticity after human prefrontal cortex damage. Neuron 68, 401–408. 10.1016/j.neuron.2010.09.018 21040843 PMC3005706

[B54] White H. R. Bray B. C. Fleming C. B. Catalano R. F. (2009). Transitions into and out of light and intermittent smoking during emerging adulthood. Nicotine Tob. Res. 11, 211–219. 10.1093/ntr/ntn017 19246434 PMC2658905

[B55] Wu G. Yang S. Zhu L. Lin F. (2015). Altered spontaneous brain activity in heavy smokers revealed by regional homogeneity. Psychopharmacology (Berl) 232, 2481–2489. 10.1007/s00213-015-3881-6 25716308

[B56] Yu D. Yuan K. Zhang B. Liu J. Dong M. Jin C.. (2016). White matter integrity in young smokers: a tract–based spatial statistics study. Addict. Biol. 21, 679–687. 10.1111/adb.12237 25752453

[B57] Yu R. Zhao L. Lu L. (2011). Regional grey and white matter changes in heavy male smokers. PLoS One 6:e27440. 10.1371/journal.pone.0027440 22076160 PMC3208641

[B58] Yu R. Zhao L. Tian J. Qin W. Wang W. Yuan K.. (2013). Regional homogeneity changes in heavy male smokers: a resting–state functional magnetic resonance imaging study. Addict. Biol. 18, 729–731. 10.1111/j.1369-1600.2011.00359.x 21812873

[B59] Yuan K. Qin W. Yu D. Bi Y. Xing L. Jin C.. (2016a). Core brain networks interactions and cognitive control in internet gaming disorder individuals in late adolescence/early adulthood. Brain Struct. Funct. 221, 1427–1442. 10.1007/s00429-014-0982-7 25573247

[B60] Yuan K. Yu D. Bi Y. Li Y. Guan Y. Lisu J.. (2016b). The implication of frontostriatal circuits in young smokers: a resting–state study. Hum. Brain Mapp. 37, 2013–2026. 10.1002/hbm.23153 26918784 PMC6867544

[B61] Zhang X. Salmeron B. J. Ross T. J. Geng X. Yang Y. Stein E. A. (2011). Factors underlying prefrontal and insula structural alterations in smokers. Neuroimage 54, 42–48. 10.1016/j.neuroimage.2010.08.008 20699124 PMC2962727

